# The role of long non-coding RNAs in the pathogenesis of hereditary diseases

**DOI:** 10.1186/s12920-019-0487-6

**Published:** 2019-03-13

**Authors:** Peter Sparber, Alexandra Filatova, Mira Khantemirova, Mikhail Skoblov

**Affiliations:** 1 Research Center for Medical Genetics, Moscow, Russia; 2 Novosibirsk State University, Novosibirsk, Russia; 3 Institute of Cytology and Genetics SB RAS, Novosibirsk, Russia; 4 School of Biomedicine, Far Eastern Federal University, Vladivostok, Russia

**Keywords:** Long non-coding RNA, lncRNA, Hereditary disease, Gene regulation, Medical genetics

## Abstract

**Background:**

Thousands of long non-coding RNA (lncRNA) genes are annotated in the human genome. Recent studies showed the key role of lncRNAs in a variety of fundamental cellular processes. Dysregulation of lncRNAs can drive tumorigenesis and they are now considered to be a promising therapeutic target in cancer. However, how lncRNAs contribute to the development of hereditary diseases in human is still mostly unknown.

**Results:**

This review is focused on hereditary diseases in the pathogenesis of which long non-coding RNAs play an important role.

**Conclusions:**

Fundamental research in the field of molecular genetics of lncRNA is necessary for a more complete understanding of their significance. Future research will help translate this knowledge into clinical practice which will not only lead to an increase in the diagnostic rate but also in the future can help with the development of etiotropic treatments for hereditary diseases.

## Introduction

In 1958, Francis Crick proposed the central dogma of molecular biology in which he explained the flow of genetic information within a biological system. In the central dogma RNA acts as a simple intermediary between the DNA that carries the genetic information and the proteins that define the whole variety of biological processes in the cell. Since then our understanding of the central dogma has dramatically changed. Large international consortiums such as ENCODE (The Encyclopedia of DNA Elements) has shown that up to 80% of the genome is transcribed while only 1,5% of it is protein –coding sequences [1]. This showed that non-coding RNAs are a lot more abundant than it was considered before.

Based on the length of the transcript non-coding RNAs are divided into two groups: short and long non-coding RNAs. The first group includes well-known classes such as tRNAs, snRNAs, snoRNAs, miRNAs, piRNAs and others. The second group includes rRNAs and long non-coding RNAs (lncRNAs), which to date are very poorly functionally annotated.

LncRNAs are defined as transcripts of more than 200 nucleotides in length not containing an extended open reading frame. Based on the annotations of the FANTOM5 project the human genome contains almost 28,000 lncRNA genes, a number that is comparable to the amount of protein-coding genes [2] . In-depth analysis revealed that lncRNAs is an extensive and very heterogenic group from mRNA-like transcripts to circular RNAs. Their length can vary from several hundred nucleotides for BC-200 to 90 thousands like in the case of Kcnqot1. LncRNA are found in all branches of life and the complexity of different organisms is well correlated with the amount and the diversity of these transcripts [3]. According to the GENCODE project one third of all human lncRNAs genes are primate-specific [4].

LncRNAs share a lot of common features with mRNAs, they are often capped, polyadenylated and undergo splicing. Nevertheless, lncRNA has its own peculiarities [4]. On average lncRNAs have less exons in their structure. 42% of lncRNA contains two exons in comparison with only 6% in mRNAs. In addition, on average lncRNA are shorter than mRNA. Five hundred ninety-two nucleotides compared to 2453 for mRNA. Whereas 95% of all multi-exon mRNAs are alternatively spliced only 25% of lncRNAs undergo alternative splicing. Generally, lncRNA has a lower expression level, their expression is more tissue-specific, and the majority of lncRNA has nuclear localization. LncRNA is less conserved than mRNA, but the sequence conservation in lncRNA is not always correlated to their function. For example, XIST (X-inactive specific transcript), one of the first described lncRNAs has a low level of sequence conservation, but a highly conserved function across placental mammals – inactivation of the X chromosome [5]. On the other hand, MALAT1 whose sequence is highly conserved between human and mice do not tend to be conserved on the functional level. Knockdown experiments on human and mice lung cancer cell lines showed a decrease migration and metastatic rate in human. On the other hand in mice knockdown of MALAT1 apparently do not lead to a distinct phenotype [6]. These examples demonstrate that for lncRNA the sequence conservation is not always a predictor for functionality, and that functional conservation may have a more complex nature in lncRNAs.

So far, only a small amount of lncRNAs is functionally annotated [7, 8]. However various studies showed that lncRNAs can participate in many cellular processes, including regulation of gene expression at the transcriptional [9–11] and post transcriptional levels [12–14]. Moreover, it is known that many lncRNAs can contribute to the development of many human diseases. Currently several databases containing entries about the association of non-coding RNAs and human diseases are available such as Lnc2Cancer, MNDR, LncRNADisease. For example, the last update of LncRNADisease (from july 26, 2017) contains almost 2000 entries about 914 lncRNAs that are associated with 329 diseases. Different approaches can be used for investigating the role of lncRNAs in the pathogenesis of human diseases. For some lncRNAs the connection with human diseases was made through GWAS (genome wide association studies) or investigating differential gene expression, but the molecular mechanisms are still unknown. For others several aspects of the pathogenesis were experimentally investigated in-vitro. Finally for some lncRNAs their role in disease progression was obtained during in-vivo experiments on mice models and in some cases lncRNAs were suggested as therapeutic targets. This review presents an analysis of the papers pertaining to each of the groups described above.

### Analysis of lncRNA association with hereditary diseases

Several approaches are used for investigating the role of lncRNA in the development of human diseases. One of them is genome wide association studies – GWAS. In this case, single nucleotide polymorphisms (SNP), associated with a particular condition are investigated. To date there are only a few examples of using GWAS in the field of lncRNA analysis. The reason is that GWAS were focused primarily on protein-coding genes [15], despite the fact that most SNPs are located outside of protein-coding regions of the genome [16]. One of the last researches that used GWAS revealed almost 150 lncRNAs associated with the cardiometabolic phenotype [15]. More detailed analysis was performed for lncRNA linc-NFE2L3–1 and identified a possible molecular mechanism by which linc-NFE2L3–1 can contribute to the development of the investigated phenotype. Another example of using GWAS analysis for identification of possible lncRNA association with human diseases is research conducted into the DISC genomic locus in the field of psychiatric disorders such as schizophrenia, bipolar disorder, depression and autism spectrum disorders. This study revealed that a protein-coding gene *DICS1* is associated with these conditions*.* This genomiс locus also contains a lncRNA gene *DICS2,* that is transcribed from the opposite strand and may possibly regulate *DICS1* expression [17]. However, there is no experimental data confirming this hypothesis and the role of *DICS2* in the development of schizophrenia.

Another approach for investigating the role of lncRNA in the development of human disease is differential expression analysis both in normal conditions and in disease state. Many papers focusing on protein-coding genes showed that the change in gene expression might be the main event in disease pathogenesis or it can be a secondary incident. Although the first situation is more interesting for studying the molecular mechanisms of disease pathogenesis, in both cases the altered expression level can be used as a very important biomarker of the pathological processes.

An example of such a work is a study of gene expression in hereditary hemorrhagic telangiectasia, also known as Osler–Weber–Rendu disease (OMIM 187300) [18]. Using microarrays the expression level of RNA extracted from normal and affected tissues of patients with Osler–Weber–Rendu disease was obtained. As a result, 42 differentially expressed lncRNAs were identified. In their work, the authors note that further study is necessary to establish the role of these lncRNAs in disease progression. However, the expression level of these genes can already be used as a diagnostic biomarker. Another work, emphasizing the importance of lncRNAs in human disease is a recently published paper investigating autism spectrum disorder (ASD) [19]. Authors compared RNA-seq data of samples from affected and control brain samples. 60 lncRNAs with a dramatically altered expression level were identified in autism. Moreover, authors showed that most of these lncRNAs are expressed in the brain and are primate-specific. Weighted gene co-expression network analysis (WGCNA) revealed six gene modules associated with ASD. Superimposing these results with GWAS data showed that one of these modules was enriched with rare pathogenic variants associated with ASD. Thereby, authors conclude that lncRNAs are very promising targets in studying ASD.

LncRNA that is often used as a biomarker is H19 – one of the first described lncRNAs [20]. It is an imprinted lncRNA, 2.3 kb in length which is normally only expressed from the maternal allele. Analysis of H19 methylation is an important step in the diagnosis of Beckwith-Wiedemann and Silver-Russell syndromes especially in the case of the latter where in 60% of cases a loss of methylation in imprinting center 1 (IC) is observed. The loss of methylation leads to biallelic expression of H19 [21].

Thus, further utilization of GWAS and analysis of differential gene expression and the combination of these methods can be very useful in investigation of lncRNA associations with various pathological conditions. However, additional insight is needed to determine the true role of lncRNA in the development of human diseases.

### LncRNAs as possible players in disease pathogenesis

Besides lncRNAs that acts as biomarkers there is data about other transcripts whose role in the disease pathogenesis is more obvious, but for now their current role in the pathogenesis of hereditary diseases is still unknown or not fully understood. A widely known lncRNA NEAT1 (Nuclear-Enriched Autosomal Transcript 1) can serve as a good example. NEAT1 normally participates in the formation of nuclear structures known as paraspeckles – ribonucleoprotein particles of cell nuclei [22, 23]. A study showed that in the early stages of amyotrophic lateral sclerosis (ALS) NEAT1 expression is induced in the motor neurons. As a result the number of paraspeckles formed by NEAT1 and other proteins including TDP-43 and FUS/TLS is increased in neurons’ nuclei [24]. Pathogenic variants in *TDP-43* and *FUS/TLS* genes are associated with different forms of ALS. However, it is still unclear why NEAT1 expression in induced in the first place.

Another example is AK056155 lncRNA which was identified during differentially expressed gene screening in endothelial cells of patients with Louis Dietz syndrome and in an aortic aneurysm samples (a common complication of this syndrome [25]). In 75% of cases Louis Dietz syndrome is caused by pathogenic variants in the transforming growth factor beta-receptor 1 gene (*TGFBR 1*) which leads to increased receptor signaling. In Louis Dietz syndrome AK056155 is upregulated due to increased signal transduction of the PI3K/AKT pathway activated by TGF-β1 binding to its receptor. Yet the way increased AK056155 expression level leads to aortic aneurysms is unexplained.

A similar situation is observed in the case of BDNF-AS lncRNA. BDNF (Brain derived neurotrophic factor) is a growth factor that is necessary for maintaining growth, survival and synaptic plasticity of neurons [26]. BDNF level in the brain is often decreased in many neurological disorders including Huntington disease, wherein overexpression of BDNF in mice models leads to an improvement of cognitive function [27]. BDNF-AS is an antisense lncRNA transcribed from the opposite strand that regulates BDNF expression level by recruiting EZH2 methyltransferase, a component of PRC2 (polycomb repressive complex) to the BDNF promoter [28]. Hereby, BDNF-AS negatively regulates BDNF level which is decreased in Huntington’s disease. However, not all aspects of BDNF-AS’ role in disease pathogenesis are clear to date. And it is still an open question how expansion in *HTT* gene is related to BDNF-AS lncRNA.

BC-200 is an example of an lncRNA associated with Alzheimer’s disease. This lncRNA is 200 nucleotides in length, it is transcribed by RNA polymerase III and expressed in neurons and localized in their synaptodendrosomes [29]. BC-200 modulates translation in neuron dendrites and is associated with synaptic plasticity [30]. In Alzheimer’s disease BC-200 is dysregulated [31]. Northern blot analysis of RNA extracted from post-mortem brain samples showed that starting from 49 years BC-200 level is decreasing. At 86 years the level of BC-200 is decreased by 65%. The results are consistent with expectations because synaptic plasticity declines with age. In Alzheimer’s disease where neuronal function is seriously altered the result was opposite. In the most affected brain areas BC-200 expression level was above control level. In areas not subjected to neurodegeneration BC-200 was normally expressed. In addition, a correlation between the level of BC-200 and disease severity was revealed. The more pronounced were the signs of dementia calculated by CDR scale (Clinical Dementia Rating) – the higher the level of BC-200 according to northern blot results. Normally BC-200 is evenly distributed in neurons. However, in Alzheimer’s disease along with high expression level BC-200 subcellular localization is altered, perinuclear inclusions are formed and BC-200 level in the dendrites is reduced. It is possible that BC-200 upregulation in Alzheimer’s disease is a compensatory reaction in response to synaptic plasticity decline. At the first stages of disease progression high levels of BC-200 can compensate the degenerative changes in neurons. But at some point decompensation happens, cellular transport of BC-200 is disrupted and BC-200 starts to form perinuclear inclusions. Although, not all is clear about the role of BC-200 in the pathogenesis of Alzheimer’s disease its expression level can serve as a good diagnostic and prognostic marker.

lncRNA may also participate in the pathogenesis of fragile X syndrome – an X-linked hereditary disorder caused by a trinucleotide CGG expansion in the 5′ UTR of *FMR1* gene. Analysis of *FMR1* genomic locus revealed that besides FMR1 mRNA three more lncRNAs are transcribed from this locus: FMR5 – a sense lncRNA, which transcribed upstream from FMR1 promoter; FMR6 an antisense lncRNA overlapping with the 3′ region of FMR1 mRNA and FMR4 (FMR1-AS1) another antisense lncRNA that overlap with the CGG expansion region. All these lncRNAs have a different brain transcription pattern in premutation and full mutation conditions [32–34]. FMR5 is expressed in all conditions including normal ones. This means that its expression is independent from FMR1 expression. FMR6 is not expressed in both pre and full mutation conditions. At last, FMR4 has the same expression pattern as FMR1. Knockdown experiments showed that FMR4 possesses an antiapoptotic effect – downregulation of FMR4 in a cell culture dramatically increased the apoptotic rate [33]. However, neither knockdown nor overexpression of FMR4 had an influence on FNR1 level which indicates a lack of direct regulation between these two transcripts. All these data shows how complicated the genomic organization of FMR1 locus is. Yet the pathogenic role of the described lncRNAs in fragile X syndrome is still on the agenda.

### Mechanisms of hereditary disease pathogenesis involving lncRNA

LncRNAs can contribute to the development of hereditary diseases by different mechanisms. The most common are the following:

- Recruitment of chromatin-modifying complexes
- Antisense transcription
- Splicing regulation
- miRNA dependent mechanism
- RNA-RNA duplex formation

The most common mechanism is recruitment of chromatin-modifying complexes. LncRNA can bind to components of repressive complexes like KCNQ1OT1 [35] that binds with PRC2.On the other hand, lncRNA can interact with activation complexes like DBE-T in facioscapulohumeral muscular dystrophy progression [36]. This mechanism of transcriptional regulation can take place not only at the origin of lncRNA transcription but can also regulate the expression of genes located in another genomic locus [10].

Another mechanism is antisense transcription of lncRNA. In this case the target gene repression occurs when lncRNA is being transcribed from the opposite strand. The transcription of antisense lncRNA is necessary but not sufficient for sense gene repression. Experiments with premature termination of lncRNA transcription showed that repression is not observed if transcription does not occur along the entire target gene. This mechanism is being implemented in Angelman syndrome [37], pseudohypoparathyroidism type 1b [38], spinocerebellar ataxia type 7 [39] and others.

LncRNA can also contribute to disease progression by splicing regulation. Some lncRNA like sno-lncRNA for instance can bind to splicing regulating factors. Their absence changes the normal ratio of these factors and this leads to the development of disease [40].On the contrary, it is the interaction of lncRNA with different splicing factors that can lead to pathology. The main event in the pathogenesis of spinocerebellar ataxia type 8 is binding of ATXN8OS to alternative splicing factor MBLN1. As a result – its subcellular localization is disrupted [41]. Change in normal splicing pattern with the participation of lncRNA is also observed in other diseases, particularly in Alzheimer’s disease [42].

lncRNAs can regulate their target gene expression by a miRNA dependent mechanisms. On one hand, lncRNA can act as a miRNA “sponge” and subsequently lead to a positive regulation of miRNA targets. Thus, the excess or lack of lncRNA can lead to the dysregulation of miRNA targets which in turn can be the cause of the pathologic processes like in the case of Hirschsprung disease [43]. On the other hand, lncRNAs themselves can be precursors of miRNA and pathogenic variants in this lncRNA can disrupt the biogenesis of miRNA. This mechanism is involved in cartilage-hair hypoplasia [44].

An example of post-transcriptional regulation mechanism is RNA-RNA duplex formation. Duplex formation can both repress translation and stabilize the mRNA protecting it from degradation and this way positively regulating its level in the cell. This mechanism takes place in Alzheimer’s disease where BACE1-AS binds to BACE1 mRNA which leads to beta-secretase upregulation. As a result – the amount of amyloid beta is increased [45].

### Well studied lncRNA and their role in hereditary disease pathogenesis

The last group are lncRNAs whose role in the progression of hereditary disease is established. Therefore this group is of the greatest interest. To date 15 hereditary diseases can be included in this group in the pathogenesis of which lncRNA plays an important role (Table 1.)

**Table 1:** LncRNA participating in the pathogenesis of human hereditary diseases

Disease	LncRNA	Mechanism	reference
1. Angelman syndrome	UBE3A-ATS	Antisense transcription	[37]
2. Prader-willi syndrome	sno-lncRNAs	Splicing regulation	[40]
3. Beckwith-Wiedemann syndrome	KCNQ1OT1	Recruitment of chromatin-modifying complexes	[36]; [59]
4. Silver-Russell syndrome	H19	Recruitment of chromatin-modifying complexes; miRNA dependent mechanism	[51–58]
5.Pseudohypoparathyroidism type 1b	A\S-1	Antisense transcription	[63, 64]
	A\B	Antisense transcription	[61, 62]
6. Alzheimer disease	BACE1-AS	RNA-RNA duplex formation	[45]
	LPR1-AS	Recruitment of chromatin-modifying complexes	[68]
	51А	Splicing regulation	[42]
	17А	Splicing regulation	[73]
	ciRS −7	miRNA dependent mechanism	[75–77]
7. Huntington disease	HTTAS	miRNA dependent mechanism	[79]
8. Spinocerebellar ataxia type 7	SCAANT1	Antisense transcription	[39]
	lnc-SCA7	miRNA dependent mechanism	[83]
9. Spinocerebellar ataxia type 8	ATXN8OS	Splicing regulation	[41]
10. Facioscapulohumeral muscular dystrophy	DBE-T	Recruitment of chromatin-modifying complexes	[36]
11. Spinal muscular atrophy	SMN-AS1	Recruitment of chromatin-modifying complexes	[97]
12. Alpha thalassemia	LUC7	Antisense transcription	[99]
13. Opitz-Kaveggia syndrome	ncRNA-a	Recruitment of chromatin-modifying complexes	[102]
14. Hirschsprung disease	cir-ZNF609	miRNA dependent mechanism	[43]
15. Cartilage-hair hypoplasia	RMRP	miRNA dependent mechanism	[44]

### Angelman syndrome (OMIM 105830)

**Fig. 1:**
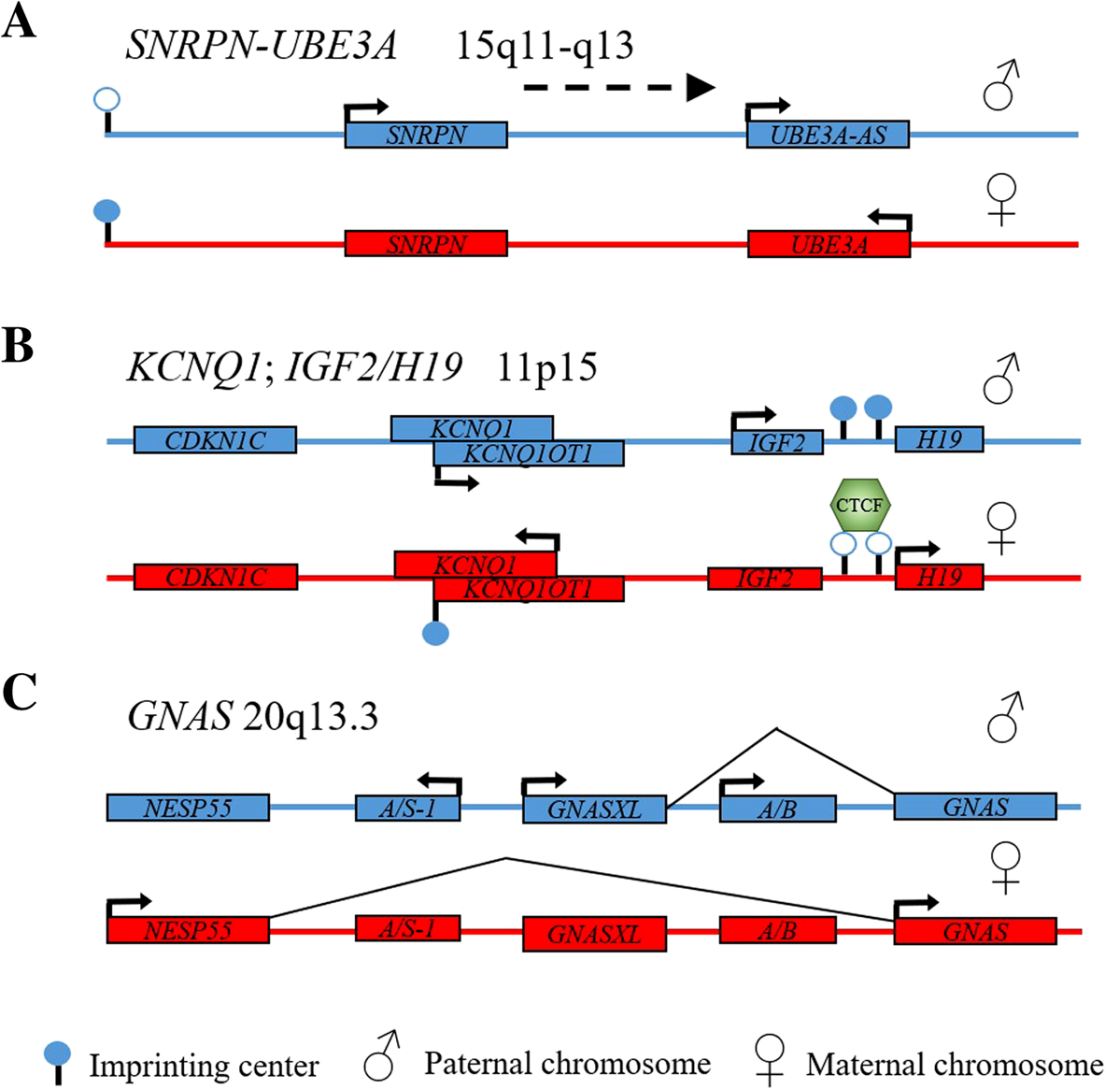
The organization and normal expression pattern of imprinted regions. Arrows indicate active promoters and direction of transcription. The paternal chromosome is marked in blue, in red – the maternal. Methylated imprinting center is indicated by a shaded circle. **a** - SNRPN-UBE3A region 15q11-q13. **b** – KCNQ1; IGF2/H19 region 11p15. **c** – GNAS region20q13.3

Angelman syndrome is a complex imprinting disorder characterized by intellectual disability, severe speech impairment, seizures and specific excitable demeanor. The cause of this syndrome in 60–70% of cases is deletion of 15q.11–13 region on the maternal chromosome (Fig. 1a). Other less frequent causes is uniparental disomy of paternal origin (2–5%) and pathogenic variants in *UBE3A* gene (20%). All these mechanisms eventually lead to a lack of expression of *UBE3A* gene that encodes for a E3 ubiquitin-ligase which is normally expressed in neurons only from the maternal allele.

Recent studies discovered UBE3A-ATS – an antisense lncRNA transcribed from the opposite strand. UBE3A-ATS is a part of a bigger transcript whose transcription start site (TSS) is located upstream to the imprinting center (IC) [46]. Yamasaki et al. showed in their work that UBE3A-ATS is expressed only from the paternal allele, the one in which the sense gene is imprinted [47]. This reciprocal expression pattern allowed to make an assumption about the direct participation of UBE3A-ATS in suppressing the transcription of UBE3A on the paternal chromosome.

The hypothesis was experimentally confirmed using a mouse model [37]. Authors showed that deletion of the UBE3A-ATS promoter leads to the activation of UBE3A on the paternal chromosome. To confirm that the transcription of UBE3A-ATS is necessary for the repression of UBE3A an experiment in mouse derived neurons containing premature termination signals for UBE3A-ATS on the paternal chromosome was performed. In this model UBE3A-ATS level was decreased whereas UBE3A expression level was increased.

In 2015 a new paper was published in which a promising therapeutic approach was developed and tested in-vivo on a mice model of Angelman syndrome. Using antisense oligonucleotide (ASO) against UBE3A-ATS authors tried to activate the expression of UBE3A from the intact paternal allele. Eventually, UBE3A expression has reached 66–90% level in-vitro and 30–50% in-vivo compering to the wild-type (WT) UBE3A expression level. Moreover, in-vivo experiments in mice showed that ASO treatment ameliorated some disease-associated symptoms [48].

### Prader-willi syndrome (OMIM 176270)

Another imprinting disorder that is caused by deletion of the same 15q11–13 region but on the paternal chromosome is Prader-willi syndrome. Obesity, intellectual disability, hypotonia and hypogonadism are often observed in this syndrome. The minimal deletion region described in Prader-willi patients spans 108 kb and contains a cluster of small nucleolar RNAs (snoRNAs) SNORD116 [49]. snoRNAs are small RNA that are involved in tRNA, rRNA and snRNA modification. Analyzing the non-polyadenylated RNA fraction that is transcribed from SNORD116 region a new class of lncRNA was discovered – sno-lncRNAs. This lncRNAs contains snoRNA sequences from both ends. In-silico a large number of splicing factor FOX2 binding sites were predicted in sno-lncRNA sequence. FOX2 immunoprecipitation with subsequent RT-PCR analysis confirmed the direct binding of FOX2 to sno-lncRNA. The absence of sno-lncRNA was showed to alter the normal splicing pattern in cells [40].

**Fig. 2:**
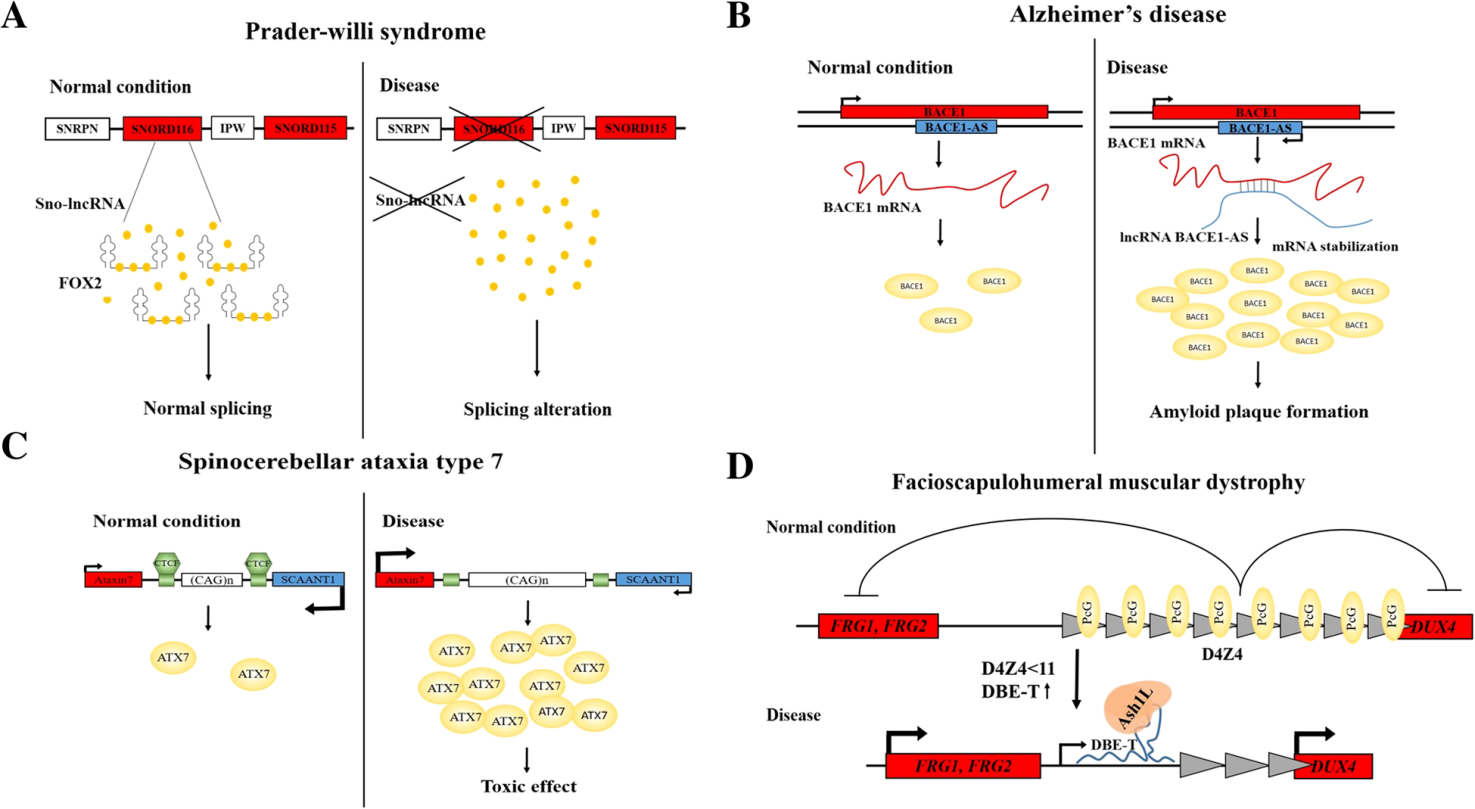
Pathogenesis schemes of inherited disease in which lncRNA participate. **a** – Deletion of SNORD116 snoRNA cluster leads to the absence of sno-lncRNA which normally binds to FOX2 splicing factor. The absence of sno-lncRNA results in excess of free FOX2 and splicing alteration leading to Prader-Willi syndrome. **b** – in Alzheimer’s disease stress conditions lead to upregulation of BACE1-AS lncRNA. BACE1-AS forms an RNA duplex with BACE1 mRNA stabilizing it. As a result the level of BACE1 is increased, which is involved in the formation of amyloid plaques leading to disease progression. **c** – CAG repeat expansion in the *Ataxin-7* gene in spinocelebellar ataxia type 7 leads to disruption of CTCF binding leading to SCAANT1 downregulation. SCAANT1 negatively regulates Ataxin-7 level and when SCAANT1 expression is decreased the level of cytotoxic Ataxin-7 is upregulated. **d** – Decrease in the number of D4Z4 repeats below 11 as a result of deletion leads to disruption of PcG binding to chromatin and transcription activation of DBE-T lncRNA occurs. DBE-T recruits Ash1L protein to chromatin, activating the transcription of nearby genes whose products possess high myopathic potential

In summary, the mechanism for Prader-Willi syndrome pathogenesis was proposed. When the SNORD116 cluster on the paternal chromosome is deleted and sno-lncRNA are absent, an excess of free FOX2 in the cells leads to a global alteration of splicing and this in turn leads to the development of disease (Fig. 2a).

### Beckwith-Wiedemann (OMIM 130650) and silver-Russell syndrome (OMIM 180860)

11p15.5 is another imprinted genomic region. Two imprinting centers (IC1 and IC2) are present in this region, both containing lncRNA genes. Imprinting disruption in the 11p15.5 region can lead to Beckwith-Wiedemann and Silver-Russell syndromes. These syndromes have opposite phenotypes: the Silver-Russell syndrome is characterized by a significant growth retardation, dwarfism and general development delay. Whereas in Beckwith-Wiedemann syndrome accelerated growth, macrosomia and an increased risk of oncological processes are the main symptoms. Wherein the main cause of Silver-Russell syndrome is hypomythelation of IC1 (37–63% of cases) in Beckwith-Wiedemann syndrome hypomythelation of IC2 (50–60% of cases) or hypermythelation of IC1(5–10% of cases) is present [50].

The first IC in 11p15.5 region is located between two genes: a protein-coding gene *IGF2* and a lncRNA gene *H19*. In normal conditions this IC is methylated on the paternal chromosome. The methylation of IC1 spreads to the *H19* promoter which represses H19 transcription. On the other hand, decreased methylation rate in IC1 leads to binding with an insulator CTCF. This binding blocks the interaction of the *IGF2* promoter with its enhancer and *IGF2* expression is decreased. Thereby, IC1 hypomethylation leads to upregulation of H19 and repression of IGF2 transcription leading to growth and developmental delay.

For investigation of the role of H19 in growth retardation transgenic mice were obtained carrying a heterozygous H19 deletion on the maternal chromosome (H19^Δ3mat/+^). These mice demonstrated elevated IGF2 levels and accelerated growth rates. However, in this condition it is difficult to distinguish in-cis effects that arise due to a change in the structure of the locus from the effects of the H19 lncRNA itself. Therefore, the authors created another mice strain expressing H19 in another genomic locus (Tg24). H19^Δ3mat/+;Tg24^ mice had a WT phenotype with a normal Igf2 expression level. This proves that the expression of H19 itself (regardless of the genomic context) leads to suppression of Igf2 expression [51]. In addition, H19 was showed to regulate the expression of several genes necessary for embryonic growth and development such as *Cdkn1c*, *Igf2r, Dcn*, *Dlk1*, *Rtl1*, *Gnas*, *Peg3 Slc38a4* [51, 52]*.* One of the mechanisms of this regulation is binding of H19 lncRNA with MBD1 protein and its recruitment to the genes functional elements. As a result, methylation of these regions occurs which leads to repression of the corresponding genes transcription [52]. Moreover, H19 can also bind to PRC2 complex – a repressive complex that inhibits transcription by histone methylation [53]. Additionally, H19 interacts with IMP-1(IGF-II mRNA-binding protein) protein, which in turn controls the translation of IGF2 mRNA and thereby regulates IGF2 on the posttranscriptional level [54]. Besides binding with proteins H19 is a precursor for miR-675 in neurons which regulates many genes by RNA interference mechanism [55, 56]. Furthermore, H19 can act as a competitive endogenous RNA (ceRNA) which binds with miRNA and thereby positively regulates its targets [57].

The second imprinting center is located in the 10 intron of *KCNQ1* gene and contains the promoter of KCNQ1OT1 lncRNA (Fig. 1b). KCNQ1OT1 is expressed only from the paternal allele. Nevertheless, in 50% of Beckwith-Wiedemann syndrome sporadic loss of methylation in IC2 is observed and KCNQ1OT1 expression becomes biallelic while the transcription of nearby genes is absent. Analyzing KCNQ1OT1 function and its role in the development of Beckwith-Wiedemann syndrome using RNA immunoprecipitation (RIP) has shown that KCNQ1OT1 binds with PRC2 complex and DNA methyltransferase Dnmt1 [58] which leads to repression of neighboring gene transcription including *Cdkn1c, Kcnq1, Slc22a18 и Phlda2*. Biallelic repression of these genes plays a key role in the development of Beckwith-Wiedemann syndrome.

### Pseudohypoparathyroidism type 1b (OMIM 603233)

Pseudohypoparathyroidism type 1b (PHP 1b) is an imprinting disorder involving the GNAS region of the 20 chromosome. This is a rare autosomal-dominant disease with a predominantly light course. The main clinical symptoms is tolerance to the action of parathyroid hormone in the proximal tubules of the kidney, with its elevation in blood, which is accompanied by a decrease in the level of calcium and an increase in the level of phosphorus in the blood plasma. The molecular basis of PHP is the absence of *GNAS* gene expression, which encodes for the stimulatory alpha subunit of a G protein (Gsα). Gsα is necessary for signal transduction of parathyroid hormone in the proximal tubule in the kidney. When Gsα is missing tolerance to parathyroid hormone develops which leads to PHP. *GNAS* is expressed from both alleles in most tissues, however in the proximal tubules, thyroid gland and in the pituitary *GNAS* is expressed only from the maternal allele [59, 60].

GNAS region includes three overlapping, imprinted protein-coding genes (*GNAS*, *GNAS-XL*, *NESP55)* and two lncRNA genes (*A\S-1* (*Nespas* in mice), *A\B)* (Fig. 1c). The lncRNA genes are also imprinted and expressed only from the paternal chromosome. Experiments in a mouse model revealed that transcription of A/B lncRNA represses the expression of GNAS in-cis [61, 62]. A/S-1 is transcribed in the antisense direction with respect to the *NESP55* gene and is also involved in repression of NESP55 in-cis [63]. Previous works have shown that *NESP55* transcription in mice oocytes leads to the establishment of the methylation status of nearby genes, in particular A\S-1 and A\B [38]. At the same time NESP55 premature transcription termination did not lead to this effect. It means that the transcription of *NESP55* is necessary to establish a normal pattern of methylation of neighboring genes.

The role of lncRNA in the development of PHP 1b was investigated in the works of Chillambhi et al. where a deletion of exons 3 and 4 of A/S1 lncRNA on the maternal chromosome was described in a patient with PHP 1b [64]. Analysis of the methylation status of gene promoters in the GNAS region using combined bisulfite restriction analysis (COBRA) showed that as a result of this deletion methylation of the *NESP55* gene promoter on the maternal chromosome occurs. At the same time A\S-1 transcription activation was observed. Thus, deletion of exons 3 and 4 of A/S1 lncRNA leads to the activation of A/S-1 transcription on the maternal chromosome. A/S-1 transcription represses NESP55 and as a result in oocytes the promoter of A/B gene is not being methylated. Later, biallelic expression of A/B leads to repression of the *GNAS* gene in the kidney tubules and the clinical picture of PHP 1b develops.

### Alzheimer’s disease (OMIM 104300)

Alzheimer’s disease – the most common form of age-related dementia – a neurodegenerative disease characterized by impaired memory, speech, and cognitive decline. The disease is developed due to neuron damage caused by accumulation of amyloid beta and aggregation of tau protein inside the cells. There is a familial form of Alzheimer’s disease caused by heterozygous pathogenic variants in *APP* gene which encodes for amyloid precursor protein. The formation of amyloid plaques in Alzheimer’s disease starts with hydrolysis of the APP protein in its beta site by the protease BACE1 (Beta-secretase 1). Subsequent cleavage by the gamma-secretase leads to the formation of amyloid beta whose oligomers form the amyloid plaque.

From the antisense strand of the BACE1 locus (11q23.3) a conservative lncRNA BACE1-AS is transcribed. BACE1-AS contains a region of 106 nucleotides that is fully complementary to exon six of BACE1 mRNA. In the work of Faghihi et al. the mechanism of BACE1-AS regulation and its role in Alzheimer’s disease was investigated [45]. Authors showed that BACE1-AS knockdown decreased not only the lever of BACE1-AS itself, but also led to BACE1 downregulation. BACE1-AS overexpression led to upregulation of BACE1 on both the RNA and the protein level. These observations clearly indicated a mechanism of positive antisense regulation. RNAse protection assay (RPA) proved that BACE1 and BACE1-AS forms a RNA duplex which stabilizes BACE1 mRNA.

It is known that various cellular stress condition such as oxidative stress plays an important role in Alzheimer’s disease [65]. Different exposures of HEK-SW cells to hyperthermia, serum fasting, high H2O2 content leads to an increase in the expression level of both BACE1 mRNA and BACE1-AS lncRNA. In addition, it was shown that the presence of amyloid beta itself in the extracellular environment leads to an increase in the level of BACE1-AS.

Expression analysis of the affected brain areas in a post-mortem sample of patients with Alzheimer’s disease revealed a 2–6 fold increase in the level of both BACE1 and BACE1-AS, compared to age-related controls. Thus, in Alzheimer’s disease, cellular stresses lead to an increase in BACE1-AS expression, which stabilizes BACE1 mRNA and increases the level of beta-secretase this results in more amyloid plaque formation which further increases the level of BACE1-AS closing the vicious circle (Fig. 2b).

LRP1 (Low-density lipoprotein receptor-related protein 1) is a protein that is involved in the pathogenesis of Alzheimer’s disease. Recent works showed that along with the regulation of cholesterol transport, this protein participates in the systemic clearance of beta-amyloid and that low LRP1 expression level is associated with the disease progression [66, 67]. In the work of Yamanaka Y et al. an antisense lncRNA LPR1-AS was discovered to negatively regulate LRP1 level [68]. RNA-Affinity chromatography with in-vitro transcribed full-length LRP1-AS showed that this lncRNA directly binds to chromatin-associated protein HMGB2. Moreover, LRP1-AS and LRP1 mRNA form an RNA-RNA duplex and the HMGB2 binding site located in the overlapping region of the two transcripts. It is known that the HMGB2 protein binds to SREBPs (Sterol regulatory element-binding proteins) [69] a family of transcription factors that regulate the expression of genes involved in lipid metabolism [70] and *LRP1* is one of them. The interaction between LRP1-AS and HMGB2 disrupts SREBP-dependent transcription of *LRP1*. As a result LRP1 level is decreased as observed in Alzheimer’s disease. LRP1-AS knockdown as well as blockage of its binding site using ASO leads to upregulation of LRP1. This can be used in the future as a therapeutic approach in Alzheimer’s disease treatment.

Another participant in Alzheimer’s disease pathogenesis is SORL1 (Sortilin-related receptor 1) protein. SORL1 level is decreased in brain samples of patients with Alzheimer’s disease and knockout of *SorLA* leads to amyloid plaque accumulation in mice brains [71]. SORL protein interacts with APP protein and regulates its cellular transport and proteolysis [71, 72]. Although the detailed mechanism remains unclarified there is convincing evidence showing that a decrease in SORL expression can lead to the development of Alzheimer’s disease. Later, an antisense lncRNA 51A was detected in this locus. It has been demonstrated that it affects the splicing of SORL1 pre-mRNA by reducing the level of the canonical isoform A but not affecting the expression level of the B and F isoforms which in turn leads to a decrease in the level of the full-length SORL protein. In addition, 51A overexpression in neuronal cell lines leads to amyloid-beta accumulation [42].

17A – a lncRNA that is transcribed from the *GABBR2* gene locus also plays a role in the development of Alzheimer disease. *GABBR2* encodes for a subunit 2 of gamma-aminobutyric acid type B receptor. 17A similarly to 51A affects GABBR2 pre-mRNA splicing, decreasing the level of the full-length protein isoform which in turn reduces GABAergic signaling in the brain. Moreover, 17A overexpression increases amyloid beta secretion in a human neuroblastoma cell line [73].

Last but not least circular RNA ciRS-7 (circular RNA sponge for miR-7) also known as CDR1-AS (cerebellar degeneration related antigen 1-antisense) plays a role in Alzheimer’s disease by miRNA-dependent mechanism. ciRS-7 is highly expressed in the brain [74] and in sporadic Alzheimer’s disease ciRS-7 expression level is reduced in the cortex and in the hippocampus [75]. This is associated with an elevated level of miR-7 for which ciRS-7 acts as a “sponge” [76]. High miR-7 level leads to downregulation of its targets which are involved in ubiquitin-dependent amyloid-beta degradation [75]. Also in-vitro experiments showed that ciRS-7 promotes both proteasome and lysosomal degradation of BACE1 and APP thereby reducing amyloid-beta levels [77].

### Huntington’s disease (OMIM 143100)

Huntington’s disease is an autosomal dominant progressive neurodegenerative disease with a late onset manifested by extrapyramidal symptoms and cognitive impairment. The expansion of CAG repeat in *HTT* gene drives translation of a mutant protein with an enlarged polyglutamine tract with cytotoxic effect which leads to disease. At normal conditions the number of repeats varies from 9 to 36 and in patients the number of CAG repeats is more than 37 [78].

HTTAS (huntingtin antisense) is an lncRNA that is transcribed from the opposite strand to *HTT* gene [79]. RACE-PCR (Rapid amplification of cDNA ends) analysis showed that there are two isoforms of this non-coding RNA of which HTTAS_v1 is of the greatest interest. The first exon of this non-coding RNA contains the CAG repeats region. Knockdown and overexpression of HTTAS_v1 demonstrated a negative regulation of HTT mRNA. Therefore, when the expression of HTTAS_v1 decreases an increase in HTT expression occurs which leads to disease aggravation. In addition, it was shown that the expression level of the antisense RNA depends on the length of the CAG repeat: the greater the number of repeats – the lower the expression. Moreover, authors showed that negative regulation of HTT is partially Dicer dependent. Experiments in dicer depleted mice stem cells overexpressing HTTAS_v1 showed no change in HTT level. Nevertheless, when increasing the CAG repeats number this effect was nullified.

### Spinocerebellar ataxia type 7 (OMIM 164500)

Spinocerebellar ataxia type 7 (SCA7) is an autosomal-dominant hereditary disease characterized by progressive cerebellar ataxia and pigmentary degeneration of the retina. SCA7 typically has late onset and the clinical signs can be very variable. SCA7 is also a repeat expansion disorder caused by CAG expansion in the *ATXN7* gene, located on the short arm of chromosome 3 (3p14). *ATXN7* encodes for the Ataxin-7 protein that normally contains a polyglutamine tract of 4–35 amino acids in length in normal conditions and 37–400 during disease state [80]. Ataxin-7 is an important component of the transcription coactivation complex STAGA. An increased polyglutamine tract length decreases STAGA complex activity which is associated with SCA7 [81].

The CAG repeat is located in the first coding exon of *ATXN7* – exon 3 which is flanked from both sides by CTCF insulator binding sites [82]. Analyzing Ataxin-7 regulation mechanism Sopher et al. described an antisense lncRNA – SCAANT1 (spinocerebellar ataxia-7 antisense noncoding transcript 1) which overlaps with *ATXN7* alternative promoter [39]. SCAANT1 negatively regulates ATXN7 mRNA level and it was observed that patients with longer CAG repeats had lower SCAANT1 expression levels. Experiments on mice showed that the binding of CTCF is necessary for the transcription of the antisense lncRNA, substitutions in CTCF binding site dramatically decreased SCAANT1 expression.

Summarizing the results the authors proposed a mechanism for *ATNX7* regulation in normal conditions and in SCA7. When the CAG repeat has normal length binding of CTCF leads to activation of SCAANT1 transcription which in turn inhibits the expression of Ataxin-7. With an increased CAG repeat or with a decreased level of CTCF expression SCAANT1 expression declines and the level of Ataxin-7 increases which leads to clinical manifestations of SCA7 (Fig. 2c).

Another lncRNA involved in SCA7 pathogenesis is lnc-SCA7 [83]. Recent work showed that lnc-SCA7 positively regulates Ataxin-7 levels. Knockdown of lnc-SCA7 led to a decrease of Ataxin-7 and an opposite effect was observed in overexpression conditions. Based on the cytoplasmic localization of lnc-SCA7 authors suggested that lnc-SCA7, similar to other lncRNAs [84], can regulate Atxn7 on the post transcriptional level acting as ceRNA for miRNA that target Atxn7. To test this hypothesis an experiment was performed on mouse embryonic stem cells carrying a homozygote deletion of the Dicer1 (DcrΔ / Δ) gene the protein product of which is involved in the biogenesis of miRNA in mammals. Knockdown of lnc-SCA7 in these cells did not lead to a change in Atxn7 level whereas in control cells the Atxn7 level was significantly decreased. Thus, it was confirmed that Atxn7 regulation is mediated by miRNA. From all miRNAs that are expressed in the brain only miR-16 and miR-124 contain binding sites for both Atxn7 and lnc-SCA7. Inhibition and overexpression of these miRNAs showed that only miRNA-124 regulates Atxn7 and lnc-SCA7 levels. As authors showed, knockdown of lnc-SCA7 reduced not only the level of Atxn7, but also the level of pre-miR-124 which was due to a decrease in the activity of the STAGA complex where Ataxin-7 is an important component.

Thereby, when Ataxin-7 protein contains a normal polyglutamine track STAGAs complex activity is not altered and pre-miR-124 level is not changed. Lnc-SCA7 competes with Atxn7 for binding with miR-124 and thus positively regulates its level. The increased polyglutamine tract in Atxn7 disrupts the normal function of the STAGA complex in SCA7. The level of miRNA-124 decreases which leads to a significant increase in the level of both lnc-SCA7 and Atxn7 which in turn leads to the development of disease. Moreover, authors were able to explain the tissue specific lesions in SCA7 by showing that miR-124 expression is higher in the cerebellum and the retina than in other tissues. Respectively, Atxn7 regulation mechanism is more pronounced in these tissues.

### Spinocerebellar ataxia type 8 (OMIM 608768)

Spinocerebellar ataxia type 8 (SCA8) is an autosomal-dominant neurological disorder with late onset, incomplete penetrance and with the cerebellum being affected predominantly. The main clinical symptoms are spastic dysarthria, nystagmus, impaired gait and change on vibration sensitivity. SCA8 develops due to CTG expansion in the lncRNA gene *ATXN8OS*. A normal allele contains 15–50 CTG repeats whereas a pathogenic allele can contain 71 to 1300 repeats [85]. Moreover, an increase in the number of CAG repeats in the *ATXN8* gene occurs on the complementary strand. This gene encodes for a small protein which consists mostly of glutamine amino acid. Hence, the pathogenesis of SCA8 involves gain-of-function pathogenic variants on both the RNA and the protein level.

In the first place, an expansion in *ATXN8OS* was initially described. Analysis of *ATXN8OS* sequence did not reveal any potential open reading frames (ORFs) Therefore, it was proposed that the cause of SCA8 is pathogenic variants with gain-of-function on the RNA level [86]. Using transgenic mice bearing CTG repeats of increased or normal length on a bacterial artificial chromosome (BAC) it was shown that only mice with an increased CTG repeat demonstrate a neurological phenotype. Functional analysis of cerebellum neurons in affected mice showed disruption in GABAergic inhibition. A more detailed analysis showed that a small open reading frame in the *ATXN8* gene on the opposite strand in the CAG orientation could be translated. Immunohistochemistry with monoclonal antibodies showed positive inclusions only in the experimental group of mice. Thus, the role of previously undescribed ATXN8 protein in the pathogenesis of SCA8 was confirmed [87].

Daughters et al. in his work demonstrated the role of ATXN8OS lncRNA in SCA8 pathogenesis [41]. RNA-FISH with Cy5-labeled (*CAG*)_10_ probes showed the presence of ribonuclear inclusions in the cerebellum of affected patients and mice bearing BAC with increased CTG repeats. Combining RNA-FISH data with immunohistochemistry authors showed the colocalization of ATXN8OS with MBLN1 protein. MBLN1 is an alternative splicing factor whose cellular localization is disrupted in SCA8 which leads to an alteration of normal splicing pattern in the cells. One of the genes that were upregulated due to Mbln1 alteration in mice is Gabt4 a GABA transporter whose upregulation can lead to changes in GABAeric signaling similar to the changes detected in previous works. Brain samples from patients with SCA8 confirmed the high level of GABT4 in humans as well.

### Facioscapulohumeral muscular dystrophy (OMIM 158900)

Facioscapulohumeral muscular dystrophy (FSHD) is considered to be the third most common form of muscular dystrophy after Duchene muscular dystrophy and myotonic dystrophy and characterized by a progressive loss of muscular strength in the upper limb and facial muscles. In more than 95% of cases, FSHD is associated with a deletion of a 3.3 kb macrosatellite D4Z4 repeat in the subtelomeric region 4q35. The numbers of D4Z4 repeats are highly polymorphic with 11–150 repeats found in healthy individuals. The disease develops if the number of D4Z4 units drops below 11. D4Z4 repeats are normally in heterohromatic state and are associated with the repressive histone mark H3K27me which in turn is associated with the repressive PRC2 complex. In FSHD patients a decrease in histone H3K27me methylation is observed and is linked to transcription derepression of genes located in the 4q35 region [88, 89]. One of the ectopically expressed genes is *DUX4*, a transcriptional factor, whose expression in myotubes in FSHD patients leads to activation of caspase 3/7 and apoptosis [90]. In addition, the D4Z4 repeats contain the DBE (D4Z4 binding element) sequence, a site necessary for PRC2 complex binding.

DBE-T (DBE-Transcript) is an lncRNA that is transcribed from the terminal D4Z4 repeat only in FSHD patients and is associated with 4q35 genes derepression. Chip-seq in disease state showed an increase in Ash1L binding to 4q35 region, a component of TrxG complex which is a PRC2 antagonist. However, knockdown of Ash1L did not lead to changes in chromatin state in the 4q35 region. Analyzing the role of DBE-T in this process a direct interaction of DBE-T and Ash1L protein was detected [36].

Therefore, in FSHD a truncation in the number of D4Z4 repeats decreases PRC2 binding to this genomic region. As a result DBE-T transcription is activated. DBE-T binds with Ash1L and recruits it to the D4Z4 locus leading to the activation of transcription of genes in the 4q35 region, including *DUX4* which has a high myopathic potential. High level of DUX4 in FSHD patients leads to muscle cell death and disease onset (Fig. 2d).

### Spinal muscular atrophy (OMIM 253300)

Spinal muscular atrophy (SMA) is an autosomal-recessive hereditary disease characterized by progressive loss of neurons in the anterior horns of the spinal cord leading to muscle weakness and atrophy [91]. The molecular diagnosis is established when biallelic pathogenic variants, most commonly deletions of *SMN1* (Survival Motor Neuron 1), are observed [92]. SMA is classified depending on disease onset. In humans duplication of *SMN1*gene results in the formation of *SMN2* gene, whose sequence is almost identical to *SMN1*. Nevertheless, *SMN2* contains a substitution in exon 7 that disrupts an exonic splicing enhancer and leads to skipping of the seventh exon. As a result, a truncated and unstable protein is formed. Despite this, about 10–20% of SMN2 mRNA is correctly spliced and a mature protein is formed that is identical to the product of the *SMN1* gene [93, 94]. At the same time, the number of *SMN2* gene copies in the human genome can vary and an increased number of *SMN2* genes is associated with a milder phenotype [95].

SMA type 1with one or two copies of the *SMN2* gene has the most early onset and most severe phenotype often leading to death before the age of two. In SMA type 3 and 4, patients harbor three and more copies of *SMN2* gene and therefore has a more late onset and less rapid disease progression [96]. Thereby, methods aimed at increasing the endogenous level of SMN2 can lead to a significant improvement in the condition of patients with SMA.

Analyzing Chip-seq data from ENCODE project showed that the PRC2 complex is interacting with the SMN2 genomic locus. Knockdown of EZH1 аnd EZH2 components of the PRC2 complex, in patient derived fibroblasts led to a two-fold increase in SMN full-length mRNA levels [97].

Previous works showed that mouse *Smn* gene has an antisense lncRNA transcribed from the opposite strand that interacts with PRC2 [53]. Investigating the possible presence of the same lncRNA in humans a new lncRNA transcribed from the SMN locus was discovered which was called SMN-AS1 (SMN-Antisense 1). Because of the high homology between *SMN1* and *SMN2* loci it was suggested that SMN-AS1 is transcribed from both loci. SMN-AS1 level highly correlates with the amount of *SMN2* genes and RNA-FISH analysis showed that SMN-AS1 colocalize with SMN pre-mRNA in the nucleus. RIP confirmed the direct interaction of SMN-AS1 with PRC2 complex which was further validated using RNA-EMSA (RNA electrophoretic mobility shift assay). Thereby it was shown that SMN-AS1 negatively regulates SMN mRNA level recruiting the PRC2 complex.

LNA-modified ASO complementary to the binding site of SMN-AS1 to PRC2 transfected to fibroblast from patients with SMA led to almost 6 fold increase in full-length SMN mRNA. At the same time, the effect of ASO treatment was very specific and had little effect on the global interactions of PRC2 in the cell and almost did not have any influence on neighboring genes expression levels. Similar results were obtained in neurons where SMN is highly expressed. 14 days exposure of neurons to ASO against SMN-AS1 increased the level of full-length SMN mRNA by two times. At the same time, ASO treatment was not cytotoxic and had no influence on cell morphology. Using ASO targeting SMN-AS1 together with a splicing corrector the use of which leads to an increase in exon 7 inclusion in SMN2 mRNA led to an additional two-fold increase in SMN2 level in neurons derived from mice harboring only *SMN2* gene. These results demonstrated that combination of these two approaches could have a maximum therapeutic effect.

### Alpha thalassemia (OMIM 141800, 141,850)

Alpha thalassemia is a hemoglobinopathy that leads to aberrant formation of hemoglobin alpha chains in erythrocytes. The disease can be caused by pathogenic variants in *HBA1* (OMIM 141800) or *HBA2* (OMIM 141850) genes located in the telomeric region of the short arm of chromosome 16. Clinical symptoms can be very diverse from asymptomatic carriage, to severe forms with severe anemia, splenomegaly and jaundice [98]. In Tufarelli et al. a new deletion was described affecting the *HBA1* gene and 13 kb in the 3′ direction [99]. The deletion did not affect the *HBA2* gene, however the patient had clinical signs of anemia. Authors showed that despite the fact that the *HBA2* gene was intact its promoter was methylated and its expression was absent. It was shown that the deletion led to the formation of a truncated LUC7L transcript. Usually this is a protein-coding gene that is expressed from the opposite to *HBA2* gene strand. The described deletion disrupted both LUC7L transcription termination and its protein-coding potential. From a protein-coding transcript it became an lncRNA whose transcription continued until the *HBA2* gene promoter and led to its methylation and transcription repression. A transgenic mice model containing a construction with *HBA2* and *LUC7L* genes in different orientations demonstrated that only cis-antisense transcription is sufficient for HBA2 repression.

### Opiz-Kaveggia syndrome (OMIM 305450)

Opiz-Kaveggia syndrome also known as the FG syndrome is an X-linked recessive disorder whose main features are intellectual disability, hypotonia, behavior problems, distinct facial phenotype and often agenesis of the corpus callosum. Opiz-Kaveggia syndrome is associated with hemizygous pathogenic variants in the *MED12* gene located at the short arm of chromosome X (Xp13). MED12 is one of the subunits of the mediator – a multiprotein complex that functions as a transcriptional coactivator interacting with transcriptional factors and RNA polymerase II. The MED12 subunit is associated with both activation and repression of transcription [100].

There is a class of lncRNAs called activating non-coding RNA (ncRNA-a) with enhancers like function that are involved in transcription activation in-cis of neighboring genes [101]. Lei et al. demonstrated the interaction between the mediator complex and ncRNA-a and how disruption of this interaction lead to the development of Opiz-Kaveggia syndrome [102]. Decreasing the level of different transcription activators authors investigated the role of their knockdown on ncRNa-a level.

Only knockdown of the mediator MED12 subunit led to a profound decline in the level of ncRNA-a7. RIP experiments using antibodies to MED12 showed that it directly binds to ncRNA-a7, ncRNA-a1 and ncRNA-a3 that also act as cis-regulators of transcription [101]. Further experiments showed that pathogenic variants associated with Opiz-Kaveggia syndrome do not affect the mediator subunit assembly and their cooperation with each other, but reduces the binding of MED12 to ncRNA-a1 and ncRNA-a3. Chromatin immunoprecipitation (ChIP) with MED12 antibodies revealed the binding of MED12 to the promoter regions of ncRNA-a genes and their targets.

In Opiz-Kaveggia syndrome pathogenic variants in the mediator subunit MED12 disrupt the binding of mediator complex to a unique class of lncRNAs- ncRNA-a, which in normal conditions are involved in recruiting the mediator to its targets. In the absence of this interaction, the transcription of mediator target genes is repressed which leads to the development of disease.

### Hirschsprung disease (OMIM 142623)

Hirschsprung disease (HSCR) – a congenital disorder characterized by a violation of innervation of the intestine due to aganglionosis of the myenteric (Auerbach’s) and submucosal (Meissner’s) neural plexus of the colon. This condition is developed as a result of a defect in neural crest enteral cell migration along the intestine and is manifested by persistent constipation. The severity of HSCR depends on the length of the aganglionic segment of the colon. The main treatment method is surgical – resection of the aganglionic segment with a formation of a colorectal anastomosis. HSCR prevalence is approximately 1:5000 and is 4 times more frequent in men [103]. HSCR can be associated with different conditions such as Down syndrome, pathogenic variants in protein-coding genes or dysregulation of miRNA regulation cell migration and proliferation.

A recent paper demonstrated the role of circular lncRNA in HSCR pathogenesis [43]. Circular lncRNas are a class of non-coding RNAs with covalently closed loop structures without a free 5′ and 3′ ends formed as a result of back splicing or from lasso structures cleaved during intron splicing [104]. In this paper, it was shown that cir-ZNF609 acts as a ceRNA. ceRNAs contain miRNA binding sites and act as miRNAs sponges regulating their levels in the cell and thereby modulating the expression level of these miRNAs target genes.

Analysis of cir-ZNF609 expression level in HSCR tissue samples and in controls showed that cir-ZNF609 level is significantly lower in HSCR patients compared with control samples. Knockdown of cir-ZNF609 in two human cell lines had the same effect – reducing cell migration and proliferation. Overexpression led to the opposite effect. Since cir-ZNF609 is localized predominantly in the cytoplasm and also because circular RNAs often function as ceRNA [105] an in-silico prediction of miRNA binding sites was made. The results showed that miR-150-is a good candidate. In HSCR colon samples miR-150-5p was upregulated as was confirmed by an expression analysis and a luciferase reporter system demonstrated a direct interaction between miR-150-5p and cir-ZNF609.

One of miR-150-5p predicted targets is AKT3 which is involved in cell migration, differentiation, regulation of apoptosis, etc. In HSCR AKT3 is downregulated and its expression level is positively correlated with cir-ZNF609 levels. Knockdown of cir-ZNF609 decreased AKT3 expression levels and a luciferase reporter system showed that AKT3 is regulated by miR-150-5b. Moreover, a new insight into the pathogenesis of HSCR was given. In addition, the authors showed that the expression levels of cir-ZNF609 can serve as a good biomarker for the diagnosis of HSCR with sensitivity and specificity of 77 and 94%, respectively which is comparable with other commonly used methods [106].

### Cartilage-hair hypoplasia (OMIM 250250)

Cartilage-hair hypoplasia (CHH) also known as metaphyseal chondrodysplasia, McKusick type is an autosomal recessive heridetory disease. CHH is caused by homozygous or compound - heterozygous pathogenic variants in the *RMRP* gene on chromosome 9p13. This disease is characterized by metaphyseal osteochondrodysplasia resulting in shortening of the limbs and dwarfism, deformation of the thorax, legs curvature and the presence of fine, sparse hair. Other variable clinical signs include immunodeficiency, anemia and Hirschsprung’s disease.

The *RMRP* gene encodes for a 268 nucleotide long lncRNA – the RNA component of the mitochondrial RNA-processing endoribonuclease. RMRP is transcribed by RNA polymerase III in the nucleus and subsequently incorporated into the protein complex [107]. Most of RMRP remains in the nucleus and the nucleolus while the minor fraction is transported to the cytoplasm [108]. RMRP was the first described RNA whose transcription occurs in the nucleus and which is subsequently transported to the mitochondria. In the mitochondria RMRP cleaves the RNA primer necessary for replication of the leading DNA strand [109]. In addition, RMRP forms a complex with the telomerase subunit having reverse transcriptase activity and this complex is involved in the formation of double-stranded RNAs that are processed into siRNA by Dicer [110]. Despite the fact that the functions of RMRP are known, the way in which the pathogenic variants in this gene cause CHH is still unknown.

A new insight into the role of RMRP was given in the work of Rogler L.E. et al. [44]. Extracting a small RNA fraction from normal liver tissue and from liver with cirrhosis with further sequencing revealed two regions of enrichment in the RMRP sequence which means that RMRP can serve as a precursor for two miRNAs – RMRP-S1 from the 5′ region of RMRP and RMRP-S2. Northern-blot in different cell lines showed that miRNA biogenesis from RMRP is cell type specific and PAR-CLIP method (photoactivatable ribonucleoside-enhanced crosslinking and immunoprecipitation) confirmed the binding of RMRP-S1 and RMRP-S2 to the Argonaute protein, a key component of the RISC (RNA-induced silencing complex) complex.

The authors also studied the level of microRNAs in fibroblasts derived from patients with different pathogenic variants causing CHH. The presence of a frequent homozygous pathogenic variant 70A > G resulted in a decrease in the level of RMRP-S1 and RMRP-S2 by 60 and 70%, respectively, with respect to control. This means that pathogenic variants may lead to a disruption in miRNA biogenesis from lncRNA.

To determine the targets of the studied miRNAs inhibition and overexpression experiments were conducted in HEK293 cell line. The results showed that RMRP-S1 influences the expression level of 35 genes and in 82% of cases the level of expression decreased. RMRP-S2 on the other hand influenced the expression level of 902 genes 75% of which were downregulated. *PTCH2* and *SOX4* were among the genes that were downregulated. These genes are involved in maintaining a normal balance between the growth of chondrocytes and bones mineralization.

To date many pathogenic variants leading to the development of CHH have been described and not all of them lie inside the miRNA sequences. One of the most frequent pathogenic variants 70A > G does not change the sequence of microRNAs and the authors suggest that a change in the secondary structure of the lncRNA can be the reason for the defect in RMRP-S1 and RMRP-S2 formation.

## Discussion

As we can see lncRNAs can participate in the development of hereditary human diseases through various mechanisms regulating the expression levels of their targets through: recruitment of chromatin-modifying complexes, antisense transcription, splicing regulation, miRNA dependent mechanism, RNA-RNA duplex formation and others. Such a wide range of pathogenetic mechanisms allows us to make an assumption that further research in this field will expand the list of hereditary diseases in the development of which lncRNAs play an important role.

## Conclusions

To date lncRNAs have not been taken into account in the diagnosis of hereditary diseases, although there is evidence that their role in the development of the disease is often central. Fundamental research in the field of molecular genetics of lncRNA is necessary for a more complete understanding of their significance. Future research will help translate this knowledge into clinical practice which will not only lead to an increase in the diagnostic rate but also in the future can help with the development of etiotropic treatments for hereditary diseases.

## Abbreviations

ALS: amyotrophic lateral sclerosis
ASD: autism spectrum disorder
ASO: antisense oligonucleotide
BAC: bacterial artificial chromosome
BACE1: Beta-secretase 1
BDNF: Brain derived neurotrophic factor
CHH: Cartilage-hair hypoplasia
ChIP: Chromatin immunoprecipitation
COBRA: combined bisulfite restriction analysis
DBE: D4Z4 binding element
ENCODE: The Encyclopedia of DNA Elements
FSHD: Facioscapulohumeral muscular dystrophy
GWAS: genome wide association studies
HSCR: Hirschsprung disease
HTTAS: huntingtin antisense
IC: imprinting center
lncRNA: long non-coding RNA
LRP1: Low-density lipoprotein receptor-related protein 1
NEAT1: Nuclear-Enriched Autosomal Transcript 1
ORF: open reading frame
PHP 1b: Pseudohypoparathyroidism type 1b
PRC2: polycomb repressive complex 2
RACE: Rapid amplification of cDNA ends
RPA: RNAse protection assay
SCA7: Spinocerebellar ataxia type 7
SCA8: Spinocerebellar ataxia type 8
SCAANT1: spinocerebellar ataxia-7 antisense noncoding transcript 1
SMA: Spinal muscular atrophy
SMN1: Survival Motor Neuron 1
SNP: single nucleotide polymorphisms
SORL1: Sortilin-related receptor 1
SREBP: Sterol regulatory element-binding protein
TSS: transcription start site
WGCNA: Weighted gene co-expression network analysis
WT: wild-type
XIST: X-inactive specific transcript

## References

[1] Consortium EP (2012). An integrated encyclopedia of DNA elements in the human genome. Nature.

[2] Hon CC (2017). An atlas of human long non-coding RNAs with accurate 5′ ends. Nature.

[3] Mattick JS, Taft RJ, Faulkner GJ (2010). A global view of genomic information--moving beyond the gene and the master regulator. Trends Genet.

[4] Derrien T (2012). The GENCODE v7 catalog of human long noncoding RNAs: analysis of their gene structure, evolution, and expression. Genome Res.

[5] Galupa R, Heard E (2015). X-chromosome inactivation: new insights into cis and trans regulation. Curr Opin Genet Dev.

[6] Diederichs S (2014). The four dimensions of noncoding RNA conservation. Trends Genet.

[7] Kornienko AE (2016). Long non-coding RNAs display higher natural expression variation than protein-coding genes in healthy humans. Genome Biol.

[8] Quek XC (2015). lncRNAdb v2.0: expanding the reference database for functional long noncoding RNAs. Nucleic Acids Res.

[9] Ng SY, Johnson R, Stanton LW (2012). Human long non-coding RNAs promote pluripotency and neuronal differentiation by association with chromatin modifiers and transcription factors. EMBO J.

[10] Rinn JL (2007). Functional demarcation of active and silent chromatin domains in human HOX loci by noncoding RNAs. Cell.

[11] Zhao J (2008). Polycomb proteins targeted by a short repeat RNA to the mouse X chromosome. Science.

[12] Cesana M (2011). A long noncoding RNA controls muscle differentiation by functioning as a competing endogenous RNA. Cell.

[13] Gong C, Maquat LE (2011). lncRNAs transactivate STAU1-mediated mRNA decay by duplexing with 3′ UTRs via Alu elements. Nature.

[14] Yoon JH (2012). LincRNA-p21 suppresses target mRNA translation. Mol Cell.

[15] Ballantyne RL (2016). Genome-wide interrogation reveals hundreds of long intergenic noncoding RNAs that associate with cardiometabolic traits. Hum Mol Genet.

[16] Hindorff LA (2009). Potential etiologic and functional implications of genome-wide association loci for human diseases and traits. Proc Natl Acad Sci U S A.

[17] Chubb JE (2008). The DISC locus in psychiatric illness. Mol Psychiatry.

[18] Torring PM (2014). Long non-coding RNA expression profiles in hereditary haemorrhagic telangiectasia. PLoS One.

[19] Parikshak NN (2016). Genome-wide changes in lncRNA, splicing, and regional gene expression patterns in autism. Nature.

[20] Bartolomei MS, Zemel S, Tilghman SM (1991). Parental imprinting of the mouse H19 gene. Nature.

[21] Wakeling EL (2011). Silver-Russell syndrome. Arch Dis Child.

[22] Bond CS, Fox AH (2009). Paraspeckles: nuclear bodies built on long noncoding RNA. J Cell Biol.

[23] Clemson CM (2009). An architectural role for a nuclear noncoding RNA: NEAT1 RNA is essential for the structure of paraspeckles. Mol Cell.

[24] Nishimoto Y (2013). The long non-coding RNA nuclear-enriched abundant transcript 1_2 induces paraspeckle formation in the motor neuron during the early phase of amyotrophic lateral sclerosis. Mol Brain.

[25] Yu B (2015). Long noncoding RNA AK056155 involved in the development of Loeys-Dietz syndrome through AKT/PI3K signaling pathway. Int J Clin Exp Pathol.

[26] Figurov A (1996). Regulation of synaptic responses to high-frequency stimulation and LTP by neurotrophins in the hippocampus. Nature.

[27] Xie Y, Hayden MR, Xu B (2010). BDNF overexpression in the forebrain rescues Huntington’s disease phenotypes in YAC128 mice. J Neurosci.

[28] Modarresi F (2012). Inhibition of natural antisense transcripts in vivo results in gene-specific transcriptional upregulation. Nat Biotechnol.

[29] Tiedge H, Chen W, Brosius J (1993). Primary structure, neural-specific expression, and dendritic location of human BC200 RNA. J Neurosci.

[30] Wang H, Tiedge H (2004). Translational control at the synapse. Neuroscientist.

[31] Mus E, Hof PR, Tiedge H (2007). Dendritic BC200 RNA in aging and in Alzheimer's disease. Proc Natl Acad Sci U S A.

[32] Ladd PD (2007). An antisense transcript spanning the CGG repeat region of FMR1 is upregulated in premutation carriers but silenced in full mutation individuals. Hum Mol Genet.

[33] Khalil AM (2008). A novel RNA transcript with antiapoptotic function is silenced in fragile X syndrome. PLoS One.

[34] Pastori C (2014). Comprehensive analysis of the transcriptional landscape of the human FMR1 gene reveals two new long noncoding RNAs differentially expressed in fragile X syndrome and fragile X-associated tremor/ataxia syndrome. Hum Genet.

[35] Pandey RR (2008). Kcnq1ot1 antisense noncoding RNA mediates lineage-specific transcriptional silencing through chromatin-level regulation. Mol Cell.

[36] Cabianca DS (2012). A long ncRNA links copy number variation to a polycomb/trithorax epigenetic switch in FSHD muscular dystrophy. Cell.

[37] Meng L, Person RE, Beaudet AL (2012). Ube3a-ATS is an atypical RNA polymerase II transcript that represses the paternal expression of Ube3a. Hum Mol Genet.

[38] Chotalia M (2009). Transcription is required for establishment of germline methylation marks at imprinted genes. Genes Dev.

[39] Sopher BL (2011). CTCF regulates ataxin-7 expression through promotion of a convergently transcribed, antisense noncoding RNA. Neuron.

[40] Yin QF (2012). Long noncoding RNAs with snoRNA ends. Mol Cell.

[41] Daughters RS (2009). RNA gain-of-function in spinocerebellar ataxia type 8. PLoS Genet.

[42] Ciarlo E (2013). An intronic ncRNA-dependent regulation of SORL1 expression affecting Abeta formation is upregulated in post-mortem Alzheimer's disease brain samples. Dis Model Mech.

[43] Peng L (2017). Circular RNA ZNF609 functions as a competitive endogenous RNA to regulate AKT3 expression by sponging miR-150-5p in Hirschsprung's disease. Oncotarget.

[44] Rogler LE (2014). Small RNAs derived from lncRNA RNase MRP have gene-silencing activity relevant to human cartilage-hair hypoplasia. Hum Mol Genet.

[45] Faghihi MA (2008). Expression of a noncoding RNA is elevated in Alzheimer's disease and drives rapid feed-forward regulation of beta-secretase. Nat Med.

[46] Runte M (2001). The IC-SNURF-SNRPN transcript serves as a host for multiple small nucleolar RNA species and as an antisense RNA for UBE3A. Hum Mol Genet.

[47] Yamasaki K (2003). Neurons but not glial cells show reciprocal imprinting of sense and antisense transcripts of Ube3a. Hum Mol Genet.

[48] Meng L (2015). Towards a therapy for Angelman syndrome by targeting a long non-coding RNA. Nature.

[49] Duker AL (2010). Paternally inherited microdeletion at 15q11.2 confirms a significant role for the SNORD116 C/D box snoRNA cluster in Prader-Willi syndrome. Eur J Hum Genet.

[50] Ounap K (2016). Silver-Russell syndrome and Beckwith-Wiedemann syndrome: opposite phenotypes with heterogeneous molecular etiology. Mol Syndromol.

[51] Gabory A (2009). H19 acts as a trans regulator of the imprinted gene network controlling growth in mice. Development.

[52] Monnier P (2013). H19 lncRNA controls gene expression of the imprinted gene network by recruiting MBD1. Proc Natl Acad Sci U S A.

[53] Zhao J (2010). Genome-wide identification of polycomb-associated RNAs by RIP-seq. Mol Cell.

[54] Runge S (2000). H19 RNA binds four molecules of insulin-like growth factor II mRNA-binding protein. J Biol Chem.

[55] Keniry A (2012). The H19 lincRNA is a developmental reservoir of miR-675 that suppresses growth and Igf1r. Nat Cell Biol.

[56] Li H (2015). miR675 upregulates long noncoding RNA H19 through activating EGR1 in human liver cancer. Oncotarget.

[57] Liu L (2016). Long noncoding RNA H19 competitively binds miR-17-5p to regulate YES1 expression in thyroid cancer. FEBS J.

[58] Mohammad F (2010). Kcnq1ot1 noncoding RNA mediates transcriptional gene silencing by interacting with Dnmt1. Development.

[59] Chen M (2009). Central nervous system imprinting of the G protein G(s)alpha and its role in metabolic regulation. Cell Metab.

[60] Plagge A, Kelsey G, Germain-Lee EL (2008). Physiological functions of the imprinted Gnas locus and its protein variants Galpha(s) and XLalpha(s) in human and mouse. J Endocrinol.

[61] Liu J (2000). A GNAS1 imprinting defect in pseudohypoparathyroidism type IB. J Clin Invest.

[62] Liu J (2000). Identification of a methylation imprint mark within the mouse Gnas locus. Mol Cell Biol.

[63] Williamson CM (2011). Uncoupling antisense-mediated silencing and DNA methylation in the imprinted Gnas cluster. PLoS Genet.

[64] Chillambhi S (2010). Deletion of the noncoding GNAS antisense transcript causes pseudohypoparathyroidism type Ib and biparental defects of GNAS methylation in cis. J Clin Endocrinol Metab.

[65] Tong Y (2005). Oxidative stress potentiates BACE1 gene expression and Abeta generation. J Neural Transm (Vienna).

[66] Deane R (2008). apoE isoform-specific disruption of amyloid beta peptide clearance from mouse brain. J Clin Invest.

[67] Holtzman DM, Herz J, Bu G (2012). Apolipoprotein E and apolipoprotein E receptors: normal biology and roles in Alzheimer disease. Cold Spring Harb Perspect Med.

[68] Yamanaka Y (2015). Antisense RNA controls LRP1 sense transcript expression through interaction with a chromatin-associated protein, HMGB2. Cell Rep.

[69] Najima Y (2005). High mobility group protein-B1 interacts with sterol regulatory element-binding proteins to enhance their DNA binding. J Biol Chem.

[70] Jeon TI, Osborne TF (2012). SREBPs: metabolic integrators in physiology and metabolism. Trends Endocrinol Metab.

[71] Andersen OM (2005). Neuronal sorting protein-related receptor sorLA/LR11 regulates processing of the amyloid precursor protein. Proc Natl Acad Sci U S A.

[72] Andersen OM (2006). Molecular dissection of the interaction between amyloid precursor protein and its neuronal trafficking receptor SorLA/LR11. Biochemistry.

[73] Massone S (2011). 17A, a novel non-coding RNA, regulates GABA B alternative splicing and signaling in response to inflammatory stimuli and in Alzheimer disease. Neurobiol Dis.

[74] Hansen TB (2011). miRNA-dependent gene silencing involving Ago2-mediated cleavage of a circular antisense RNA. EMBO J.

[75] Zhao Y (2016). Deficiency in the Ubiquitin Conjugating Enzyme UBE2A in Alzheimer's Disease (AD) is Linked to Deficits in a Natural Circular miRNA-7 Sponge (circRNA; ciRS-7). Genes (Basel).

[76] Hansen TB (2013). Natural RNA circles function as efficient microRNA sponges. Nature.

[77] Shi Z (2017). The circular RNA ciRS-7 promotes APP and BACE1 degradation in an NF-kappaB-dependent manner. FEBS J.

[78] Duyao M (1993). Trinucleotide repeat length instability and age of onset in Huntington's disease. Nat Genet.

[79] Chung DW (2011). A natural antisense transcript at the Huntington's disease repeat locus regulates HTT expression. Hum Mol Genet.

[80] Paulson HL, Bonini NM, Roth KA (2000). Polyglutamine disease and neuronal cell death. Proc Natl Acad Sci U S A.

[81] Palhan VB (2005). Polyglutamine-expanded ataxin-7 inhibits STAGA histone acetyltransferase activity to produce retinal degeneration. Proc Natl Acad Sci U S A.

[82] Filippova GN (2001). CTCF-binding sites flank CTG/CAG repeats and form a methylation-sensitive insulator at the DM1 locus. Nat Genet.

[83] Tan JY (2014). Cross-talking noncoding RNAs contribute to cell-specific neurodegeneration in SCA7. Nat Struct Mol Biol.

[84] Tay Y, Rinn J, Pandolfi PP (2014). The multilayered complexity of ceRNA crosstalk and competition. Nature.

[85] Todd PK, Paulson HL (2010). RNA-mediated neurodegeneration in repeat expansion disorders. Ann Neurol.

[86] Ranum LP, Day JW (2004). Pathogenic RNA repeats: an expanding role in genetic disease. Trends Genet.

[87] Moseley ML (2006). Bidirectional expression of CUG and CAG expansion transcripts and intranuclear polyglutamine inclusions in spinocerebellar ataxia type 8. Nat Genet.

[88] Bodega B (2009). Remodeling of the chromatin structure of the facioscapulohumeral muscular dystrophy (FSHD) locus and upregulation of FSHD-related gene 1 (FRG1) expression during human myogenic differentiation. BMC Biol.

[89] Gabellini D, Green MR, Tupler R (2002). Inappropriate gene activation in FSHD: a repressor complex binds a chromosomal repeat deleted in dystrophic muscle. Cell.

[90] Kowaljow V (2007). The DUX4 gene at the FSHD1A locus encodes a pro-apoptotic protein. Neuromuscul Disord.

[91] Wirth B (2000). An update of the mutation spectrum of the survival motor neuron gene (SMN1) in autosomal recessive spinal muscular atrophy (SMA). Hum Mutat.

[92] Lefebvre S (1995). Identification and characterization of a spinal muscular atrophy-determining gene. Cell.

[93] Lorson CL (1999). A single nucleotide in the SMN gene regulates splicing and is responsible for spinal muscular atrophy. Proc Natl Acad Sci U S A.

[94] Monani UR (1999). A single nucleotide difference that alters splicing patterns distinguishes the SMA gene SMN1 from the copy gene SMN2. Hum Mol Genet.

[95] Feldkotter M (2002). Quantitative analyses of SMN1 and SMN2 based on real-time lightCycler PCR: fast and highly reliable carrier testing and prediction of severity of spinal muscular atrophy. Am J Hum Genet.

[96] Kolb SJ, Kissel JT (2015). Spinal muscular atrophy. Neurol Clin.

[97] Woo CJ (2017). Gene activation of SMN by selective disruption of lncRNA-mediated recruitment of PRC2 for the treatment of spinal muscular atrophy. Proc Natl Acad Sci U S A.

[98] Taher AT, Weatherall DJ, Cappellini MD (2018). Thalassaemia. Lancet.

[99] Tufarelli C (2003). Transcription of antisense RNA leading to gene silencing and methylation as a novel cause of human genetic disease. Nat Genet.

[100] Ding N (2008). Mediator links epigenetic silencing of neuronal gene expression with x-linked mental retardation. Mol Cell.

[101] Orom UA (2010). Long noncoding RNAs with enhancer-like function in human cells. Cell.

[102] Lai F (2013). Activating RNAs associate with mediator to enhance chromatin architecture and transcription. Nature.

[103] Goldberg EL (1984). An epidemiological study of Hirschsprung's disease. Int J Epidemiol.

[104] Chen LL, Yang L (2015). Regulation of circRNA biogenesis. RNA Biol.

[105] Qu S (2015). Circular RNA: a new star of noncoding RNAs. Cancer Lett.

[106] de Lorijn F (2006). Diagnostic tests in Hirschsprung disease: a systematic review. J Pediatr Gastroenterol Nutr.

[107] Chang DD, Clayton DA (1989). Mouse RNAase MRP RNA is encoded by a nuclear gene and contains a decamer sequence complementary to a conserved region of mitochondrial RNA substrate. Cell.

[108] Li K (1994). Subcellular partitioning of MRP RNA assessed by ultrastructural and biochemical analysis. J Cell Biol.

[109] Chang DD, Clayton DA (1987). A novel endoribonuclease cleaves at a priming site of mouse mitochondrial DNA replication. EMBO J.

[110] Maida Y (2009). An RNA-dependent RNA polymerase formed by TERT and the RMRP RNA. Nature.

